# Comparative analysis of contributions of wet deposition and photodegradation to the removal of atmospheric BaP by MFDCCA

**DOI:** 10.1038/s41598-021-85224-3

**Published:** 2021-03-09

**Authors:** Chunqiong Liu, Yuanyuan Guo, Kai Shi, Jiao Zhang, Bo Wu, Juan Du

**Affiliations:** 1 College of Biology and Environmental Sciences, Jishou University, Jishou, 416000 Hunan China; 2 China Environment Publishing Group, Beijing, 100000 China; 3 College of Mathematics and Statistics, Jishou University, Jishou, 416000 Hunan China

**Keywords:** Environmental sciences, Mathematics and computing

## Abstract

Benzo [a] pyrene (BaP) in the atmosphere possess great carcinogenic potential to human health, and the understanding of its scavenging mechanisms has attracted considerable attention. In this work, a new quantitative method is proposed to make a comparative analysis of the long-term contributions of wet deposition and photodegradation to BaP removal based on multi-fractal detrended cross-correlation analysis (MFDCCA). According to the precipitation and global solar radiation (GSR) observations from 1998 to 2016 for two urban sites (Central/Western District and TsuenWan) in Hong Kong, the wet deposition and photodegradation of BaP are analyzed. Using MFDCCA method, long-term cross-correlation between precipitation/GSR and BaP are investigated. Moreover, the differences of multifractal features in cross-correlations of precipitation-BaP and GSR-BaP system are analyzed. Strong long-term persistence is observed in the cross-correlations for precipitation-BaP system in a one-year cycle; while cross-correlations between GSR and BaP show weak persistence over the whole timescale. Based on the meteorology in Hong Kong, this difference has been discussed. Then, contributions of wet deposition and photodegradation to atmospheric BaP removal are quantified based on MFDCCA method, which are further compared between summer and winter. The comparative analysis suggests that wet deposition plays a more significant role in the removal of atmospheric BaP. Specifically, in summer, the contributions of wet deposition are twice as much as that of photodegradation for both two sites; while in winter, the contribution of photodegradation is a little higher than that of wet deposition to BaP removal. Meanwhile, for wet deposition, the contributions in summer are about ten times greater than that in winter; while for photodegradation, the difference in contributions between summer and winter are relatively smaller. Furthermore, based on sliding window technique, the temporal evolutions in the contributions of wet deposition/photodegradation to BaP removal have been presented for both two sites. On this basis, it is discovered that the comprehensive contributions of wet deposition and photodegradation peak in June, and reach their lowest levels in December for both two sites. Quantifying the contribution of meteorological factors to the removal of atmospheric BaP is help for understanding its geochemical cycle.

## Introduction

Benzo (a) pyrene (BaP) is mainly produced by the incomplete combustion processes, such as biofuel combustion, industrial production, vehicle exhaust, etc^1–4^. Because of its carcinogenic and mutagenic character^5–7^, BaP is a serious threat. It is often taken as an indicator of the total carcinogenic potential of atmospheric PAHs^8,9^. Therefore, more and more attention has been paid on the removal mechanism of the atmospheric BaP.

It has been reported that atmospheric BaP partition between the particle and vapor phases^10,11^, and the majority of BaP is bound to the particulate matter (PM) in atmosphere, namely PM-bound BaP^12–14^. It is accepted that precipitation scavenging has been considered as a significant removal way of PM. So it can be inferred that wet deposition may be an important removal way of PM-bound BaP^15–18^. In addition, the gas-particle partitioning of BaP in atmosphere can be influenced by some meteorological factors, for instance, the higher the temperature, the more BaP in the gas phase^19–22^. For BaP in gas phase, due to its low solubility in water, wet deposition seem to have no significant effect on its removal^10^. Instead, it is possible that photochemical degradation makes an appreciable contribution to the removal of gas BaP^23,24^. Thus it can be seen that the meteorological factor, i.e. precipitation and GSR, plays significant role in the removal of BaP in real atmosphere. By exploring the contribution of meteorological factors to the removal of atmospheric BaP, it is helpful to understand its geochemical cycle.

In the last decade, numerous studies on wet deposition and photochemical degradation of atmospheric BaP have been conducted using modeling methods^3,25–28^. However, due to the significant uncertainty in the processes of determining gas-particle partitioning and emissions inventories, the output of the models often have great uncertainty^23^. Some other studies on these two atmospheric processes have been carried out in the laboratory. Specifically, the capability of wet deposition in removal of BaP was mainly examined by collecting and analyzing rainfall samples by laboratory experiment^15–18,29^. In this way, the contribution of precipitation to BaP removal can only be quantitatively determined in a short time scale, but the analysis results based on short-term data tend to undergo large fluctuations. Due to the limitation of experimental conditions, it is difficult to assess the long-term trend (years or even more) of the removal efficiency depending on laboratory researches. Meanwhile, experimental studies on photodegradation of BaP mainly focused on the reaction mechanism by investigating the reactions of BaP with the OH radical in the laboratory^24,30^. Thus, in real atmosphere, it is difficult to quantitatively estimate the long-term trends in the contribution of wet deposition and photodegradation to BaP removal.

Based on the long-term field observation data, Garrido et al. (2014) have correlated BaP levels with meteorological parameters to determine the effect of climatic factors on the variability of BaP levels by Pearson correlation coefficient^31^. However, owing to the comprehensive influence of multiple factors, BaP level is not only related to meteorological factors, but also to regional transport and pollution sources. Therefore, Pearson correlation coefficient is not suitable to reflect the effects of meteorological factors on BaP removal, based on long-term monitoring data. Moreover, the pollutants time series show strong non-linear and non-stationary properties^32,33^. The trends that exist in the non-stationary systems tend to result in a false detection of long-range correlations. Pearson correlation coefficient, which can only be used to analyze the stationary series, seems to be incapable of eliminating the trends and exploring the correct cross-correlation between two non-stationary time series. Therefore, it is necessary to find novel research perspective to quantitatively determine the contribution of wet deposition and photodegradation to the removal of BaP in the actual atmosphere. In recent years, some nonlinear fractal methods have gradually been proposed to study the properties of non-stationary signals in real atmosphere^32,34–37^. Some of these methods can quantitatively determine the coupling correlation among different non-stationary signals in real atmosphere^38–40^. In particular, for analyzing the cross-correlations between two non-stationary time series, detrended cross-correlation analysis (DCCA) has been introduced to investigate the power law cross-correlations^41^. Later on, an advanced version of the DCCA method, namely multi-fractal detrended cross-correlation analysis (MFDCCA), has been developed to further investigate the multi-fractal behavior between two series recorded simultaneously^42^.Therefore, these methods play an important role in revealing the influence of meteorological parameters on the temporal and spatial evolution of air pollutants in real atmosphere. In the previous study, MFDCCA method was used to assess the wet deposition pathway of BaP^43^. It was found that the main removal process of atmospheric BaP was rainfall deposits of PM_2.5_-bound BaP. However, compared to wet deposition, the contributions of photodegradation to BaP removal still remain unclear.

In this work, based on 18 years regular monitoring data from Tsuen Wan and Central/Western District monitoring stations in Hong Kong, a new quantitative method has been proposed to make a comparative analysis of the long-term trend in the contributions of wet deposition and photodegradation to BaP removal using MFDCCA method.

## Results and discussion

### The long-term cross-correlations

The decay pattern of cross-correlations with time can provide important insights to understand the dynamics of interacting complex systems. For example, cross-correlation functions decays exponentially with time in a randomly forced first-order linear system, while some different decay pattern will occur in a higher order complex system. In order to confirm whether precipitation or GSR contributes to the removal of atmospheric BaP, it is necessary to test the decay pattern of cross-correlations between them by the DCCA exponent ${\text{h}}_{{{\text{xy}}}} \left( 2 \right)$.

**Figure 1:**
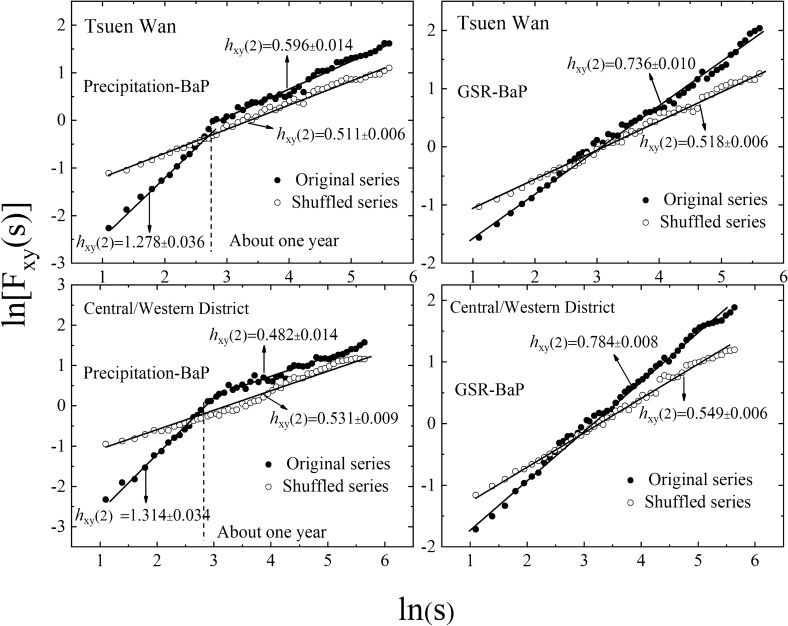
DCCA plot of precipitation/GSR and BaP for Tsuen Wan and Central/Western District sites.

According to Eq. (6), ${\text{h}}_{{{\text{xy}}}} \left( 2 \right)$ can be obtained by calculating the slope of the log–log plots of $F_{xy} \left( {q,s} \right)$ versus time scale *s,* based on 18-year observational data from 1998 to 2016. The results are shown in Fig. 1. For both Tsuen Wan and Central/Western District sites, the DCCA results of GSR-BaP exhibit clear power-law scaling relationship at the whole time scale of 18 years. The cross-correlation exponent ${\text{h}}_{{{\text{xy}}}} \left( 2 \right)$ is 0.736 and 0.784 for Tsuen Wan and Central/Western District site respectively. ${\text{h}}_{{{\text{xy}}}} \left( 2 \right) > 0.5$ function shows the long-term cross-correlations between GSR and BaP series. This special cross-correlation pattern implies that GSR fluctuations in small time scales are positively cross-correlated to BaP concentrations in the form of power-law.

Comparatively, for both Tsuen Wan and Central/Western District sites, one crossover point can be found in the DCCA curves of precipitation-BaP. The sampling frequency with about once or twice a month determines that the smallest time scale is one or half month. Since ${\text{ln}}\left( {\text{s}} \right) = 2.71$ at the crossover point, the crossover points occur at approximately one year. On one year time scale, DCCA exponent ${\text{h}}_{{{\text{xy}}}} \left( 2 \right)$ is 1.278 and 1.314 for Tsuen Wan and Central/Western District sites respectively, while over a longer time scale, both ${\text{h}}_{{{\text{xy}}}} \left( 2 \right)$ values are about 0.5. It indicates that there are stronger long-term cross-correlations between precipitation and BaP in a one-year cycle compared with that between GSR and BaP. However, in longer time scale regimes (greater than one year), there is no cross-correlation between precipitation and BaP.

The long-term cross-correlations between precipitation/GSR and BaP do not claimed the existence of special periodicities, but rather the presence of dynamical links at different time scales. The precipitation at Hong Kong is dominated by the southwest monsoon bringing humidity and rains to Asia. Thus, the precipitation mainly occurs from May to September, accounting for about 80% of the annual precipitation. According to the observations of precipitation collected once or twice a month simultaneously with BaP, the average annual precipitation for Hong Kong is 60 mm in summer, while in winter, the average annual precipitation was only about 4 mm owning to the northeast monsoon. The long-term cross-correlations between precipitation and BaP are strongly affected by the Asian monsoon system. Thus, the crossover points with about one year in the DCCA curves of precipitation-BaP may come from the annual change in the dynamical correlation properties of atmospheric circulation and regional transport. These results are in good agreement with that of previous study about dioxins^40^. At the same time, Hong Kong is located in the subtropical zone. For global solar radiation, the annual average is 49 MJ/m^2^ d, the average in summer (from June to August) is 58 MJ/m^2^ d, and the average in winter (from December to next February) is 41 MJ/m^2^ d. It can be seen that global solar radiation changes slightly in a year. Thus, there is not any crossover point in the DCCA curves of GSR-BaP owing to the absence of the dynamical correlation change in global solar radiation.

In order to test the reliability of the DCCA method, the other DCCA plots are made using randomly shuffled series as shown in Fig. 1. This is accomplished through reconstructing new shuffled series by randomly sampling from original precipitation/GSR series, which can destroy the temporal correlations in the data. Moreover, the randomly shuffling procedure has been operated twenty times, and the shuffled series are used for DCCA analysis. Figure 1 shows that the DCCA indexes ${\text{h}}_{{{\text{xy}}}} \left( 2 \right)$ for the shuffled series are all about 0.5 for the two sites, which indicate the absence of cross-correlations in randomly shuffled system. This result confirms that DCCA analysis can exactly reflect the cross-correlation between two non-stationary series.

### The multifractal features of cross-correlations

DCCA method reveals that the scaling properties of the cross-correlation between precipitation and BaP are completely different from that between GSR and BaP. Moreover, the crossover points detected by DCCA suggest that the scaling properties of the cross-correlations are more complicated, which require more scaling exponents to describe cross-correlation properties at different time scales. Multifractal approach can provide comprehensive information about inner regularity in the scaling properties of cross-correlation structures.

Based on multifractal theory, the cross-correlations between precipitation/GSR and BaP are composed of a series of intertwined monofractal subsets over different timescales. These subsets are combined into a multifractal spectrum, which can describe the heterogeneity and temporal distribution pattern of cross-correlations over the whole time scales. Therefore, taking into account the cross-correlations over different time scales, MFDCCA method is adopted to reveal the multifractal features of the cross-correlations between precipitation/GSR and BaP.

**Figure 2:**
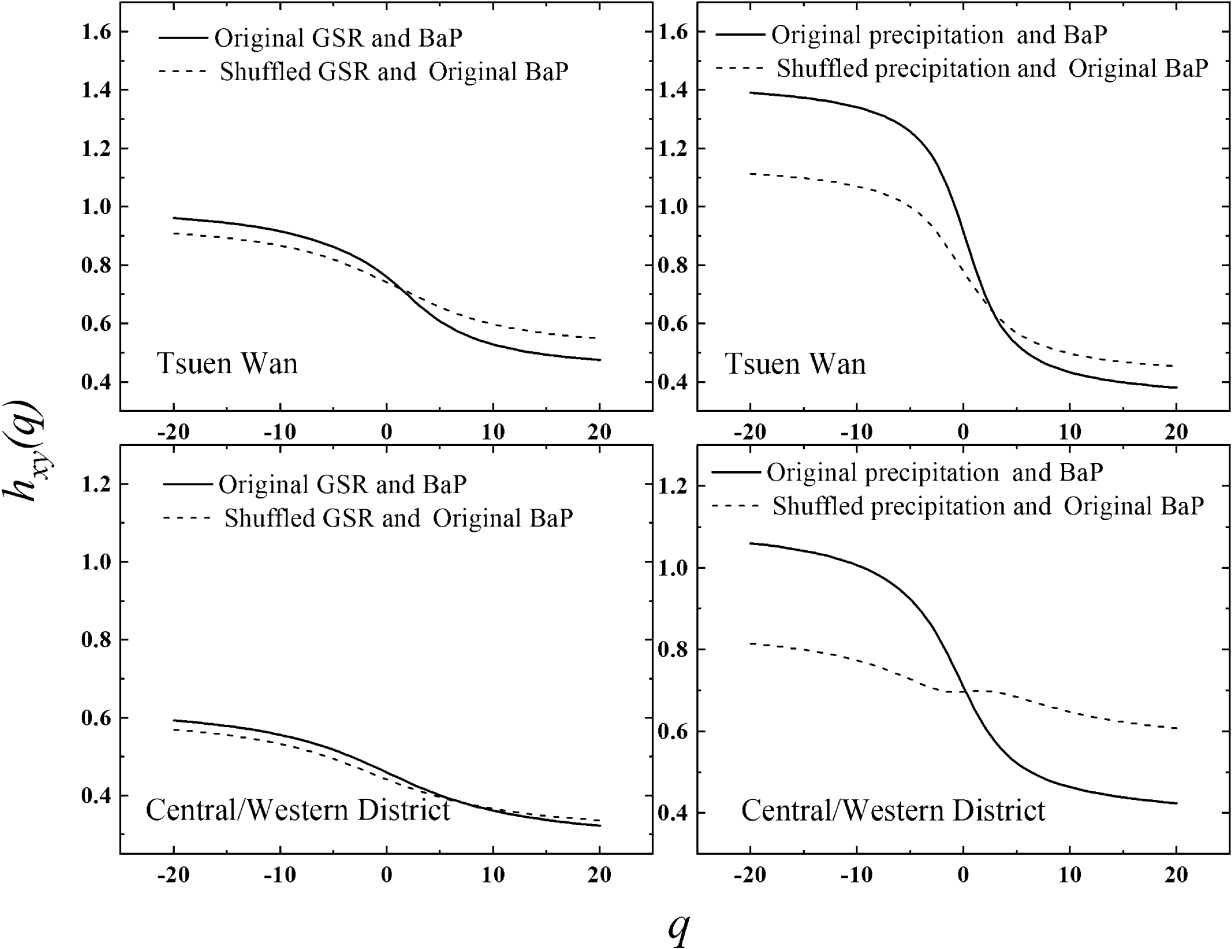
The *q* dependences of generalized cross-correlation exponent ${\text{h}}_{{{\text{xy}}}} \left( {\text{q}} \right)$ in the MFDCCA method for the two monitoring sites.

The multifractal results are displayed in Fig. 2*,* based on 18-year observational data from 1998 to 2016. For both Tsuen Wan and Central/Western District sites, the generalized cross-correlation exponents ${\text{h}}_{{{\text{xy}}}} \left( {\text{q}} \right)$ between precipitation/GSR and BaP decrease with the increase of the moments *q*. This suggests that the temporal scaling properties of the cross-correlations between precipitation/GSR and BaP are characterized by multifractality. In other words, it shows that the temporal distribution pattern of cross-correlations between precipitation/GSR and BaP has different singularity and self-similar propterties at different timescales. According to Eq. (7), $\Delta h$ means the strength of multifractality. The high $\Delta h$ values imply that the variability and heterogeneity of cross-correlations between precipitation/GSR and BaP over different timescales is powerful. In Fig. 2, for precipitation-BaP system, $\Delta h$ values are 1.01 and 0.64 for Tsuen Wan and Central/Western District sites respectively. Meanwhile, for GSR-BaP system, $\Delta h$ value is 0.5 and 0.27 for the two sites respectively. From these results, it can be seen that the multifractal degree of cross-correlations between precipitation and BaP is much stronger than that between BaP and GSR. It implies that the temporal distribution pattern of cross-correlations between BaP and precipitation are more complex compared with that between GSR and BaP. This result suggests that multifractal approach can provide significant information about comparative analysis of contributions of wet deposition and photodegradation to the removal of atmospheric BaP.

### The contribution of precipitation/GSR to the removal of atmospheric BaP

Based on 18-year observational data from 1998 to 2016, the contributions of precipitation/GSR to BaP removal have been quantified by MFDCCA. After obtaining the original $h_{xy} \left( q \right) \propto q$ as shown by the solid line in Fig. 2, the shuffling procedure is applied on the original precipitation/GSR series and kept BaP series unchanged. New ${\text{ h}}_{{{\text{xy}}}} \left( {\text{q}} \right)$ curves can be obtained for the shuffled precipitation/GSR-original BaP series based on MFDCCA as shown by the dotted line in Fig. 2. The degree of deviation between the original and shuffled $h_{xy} \left( q \right) \propto q$ curves can be quantified by $\chi_{{}}^{2}$, according to Eqs. (8) and (9). In Fig. 2, as to Tsuen Wan site, for precipitation-BaP, ${\upchi }^{2} = 2.31$, while for GSR-BaP, ${\upchi }^{2} = 1.83$. As to Central/Western District site, for precipitation-BaP, ${\upchi }^{2} = 3.43$, while for GSR-BaP, ${\upchi }^{2} = 2.69$. Obviously, the contribution of precipitation and GSR to BaP removal in Central/Western District site is greater than that in Tsuen Wan site. The results are reasonable. The ambient BaP samples were collected once or twice a month from January 1998 to December 2016 by HKEPD. According to the simultaneously observations of precipitation and GSR, the average annual precipitation in Central/Western District site is 107 mm, while that in Tsuen Wan site is 94 mm. In general, the more precipitation is, the more significant the wet deposition of BaP is, and the greater contribution of precipitation to BaP removal is. Meanwhile, the average annual GSR is 201 MJ/m^2^ d in Central/Western District site, while that in Tsuen Wan site is 199 MJ/m^2^ d. Generally, the stronger the solar radiation is, the higher the probability of BaP being converted into other components by photochemical reactions, and the greater contribution of GSR to BaP removal is.

**Figure 3:**
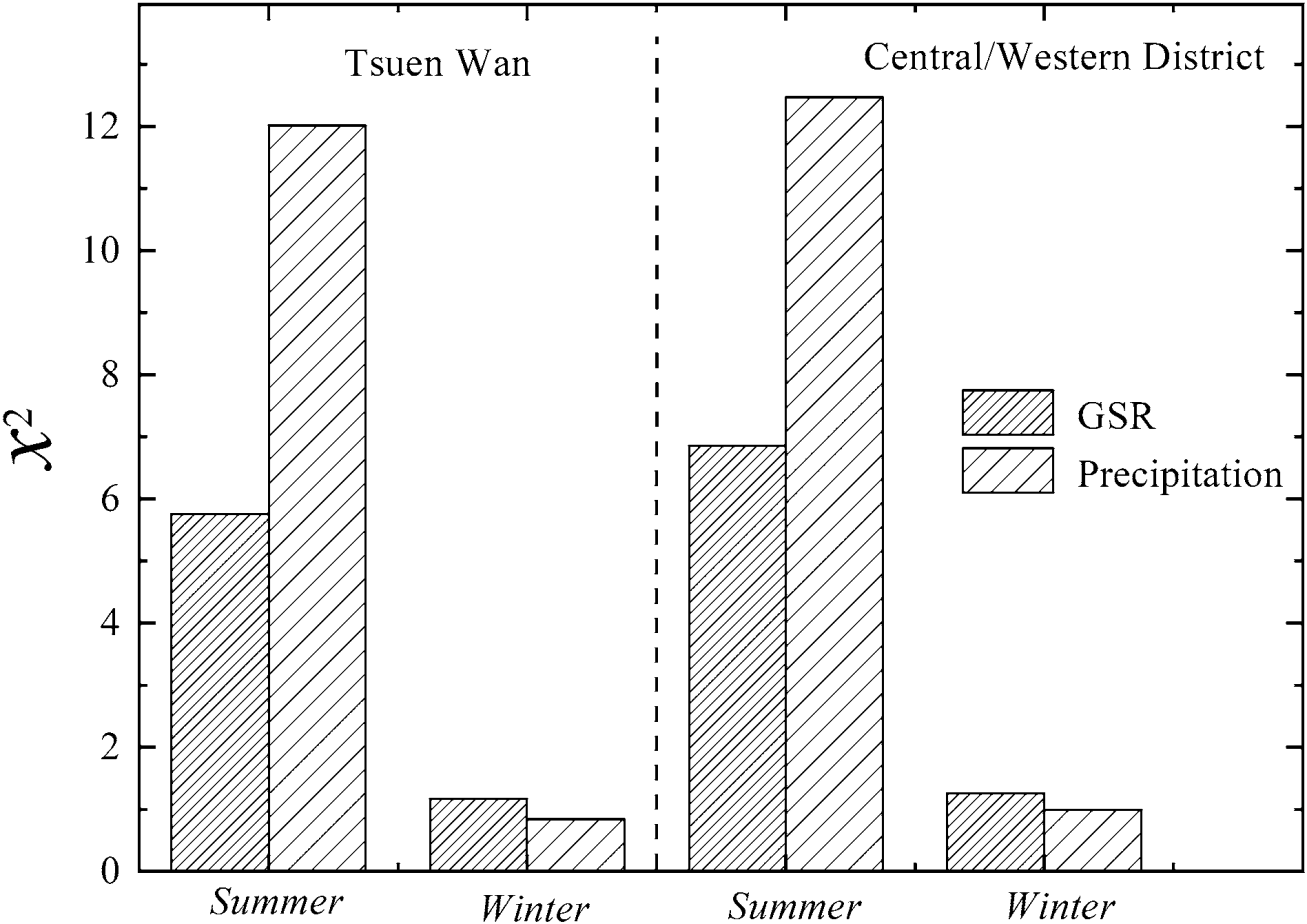
The contribution of precipitation/GSR to BaP removal in summer and winter for the two sites.

It should be noted that the above results are obtained over the whole time scale of 18 years from1998 to 2016. However, because of the influence of meteorological factors, there may be seasonal differences in the contribution of precipitation/GSR to BaP removal. So in order to test the availability of the method, we have quantified the contribution of precipitation/GSR to BaP removal by calculating the $\chi_{{}}^{2}$ values in summer (from June to August) and in winter (from December to next February) for the two sites. As shown in Fig. 3, for Central/Western District site, $\chi_{{}}^{2}$ values for precipitation (GSR) are 12.47 (6.85) and 0.99 (1.26) in summer and winter respectively; while for Tsuen Wan site, $\chi_{{}}^{2}$ values for precipitation (GSR) are 12.01 (5.75) and 0.84 (1.17) in summer and winter respectively. Two conclusions can be drawn as follows: (i) The $\chi_{{}}^{2}$ values of precipitation and GSR in summer are all higher than that in winter for the two sites. The contribution of precipitation to BaP removal in summer is more than 10 times higher than that in winter. While the contribution of GSR to BaP removal in summer is about five times higher than that in winter. (ii) In summer, the contribution of precipitation to BaP removal is about two times larger than that of GSR for both two sites. In winter, the difference in the contributions between precipitation and GSR is not very significant, and the contribution of GSR is a little higher than that of precipitation. These results can be explained by the meteorological difference between summer and winter. Hong Kong belongs to subtropical climate, with precipitation concentrated in summer. According to the simultaneously observations of precipitation and GSR, the annual average precipitation is 66 mm (Central/Western District site) and 53 mm (Tsuen Wan site) in summer respectively, but is 4 mm (Central/Western District site) and 3 mm (Tsuen Wan site) in winter respectively. Meanwhile, the annual average GSR is 59 MJ/m^2^ d (Central/Western District site) and 57 MJ/m^2^ d (Tsuen Wan site) in summer respectively, but is 41 MJ/m^2^ d (Central/Western District site) and 40 MJ/m^2^ d (Tsuen Wan site) in winter respectively. Thus, abundant precipitation and solar radiation in summer can enhance the wet deposition and photochemical reactions of BaP, promoting BaP removal from atmosphere. In winter, the inadequate precipitation and high GSR lead to the fact that the contribution of photodegradation is greater than that of wet deposition in winter.

**Figure 4:**
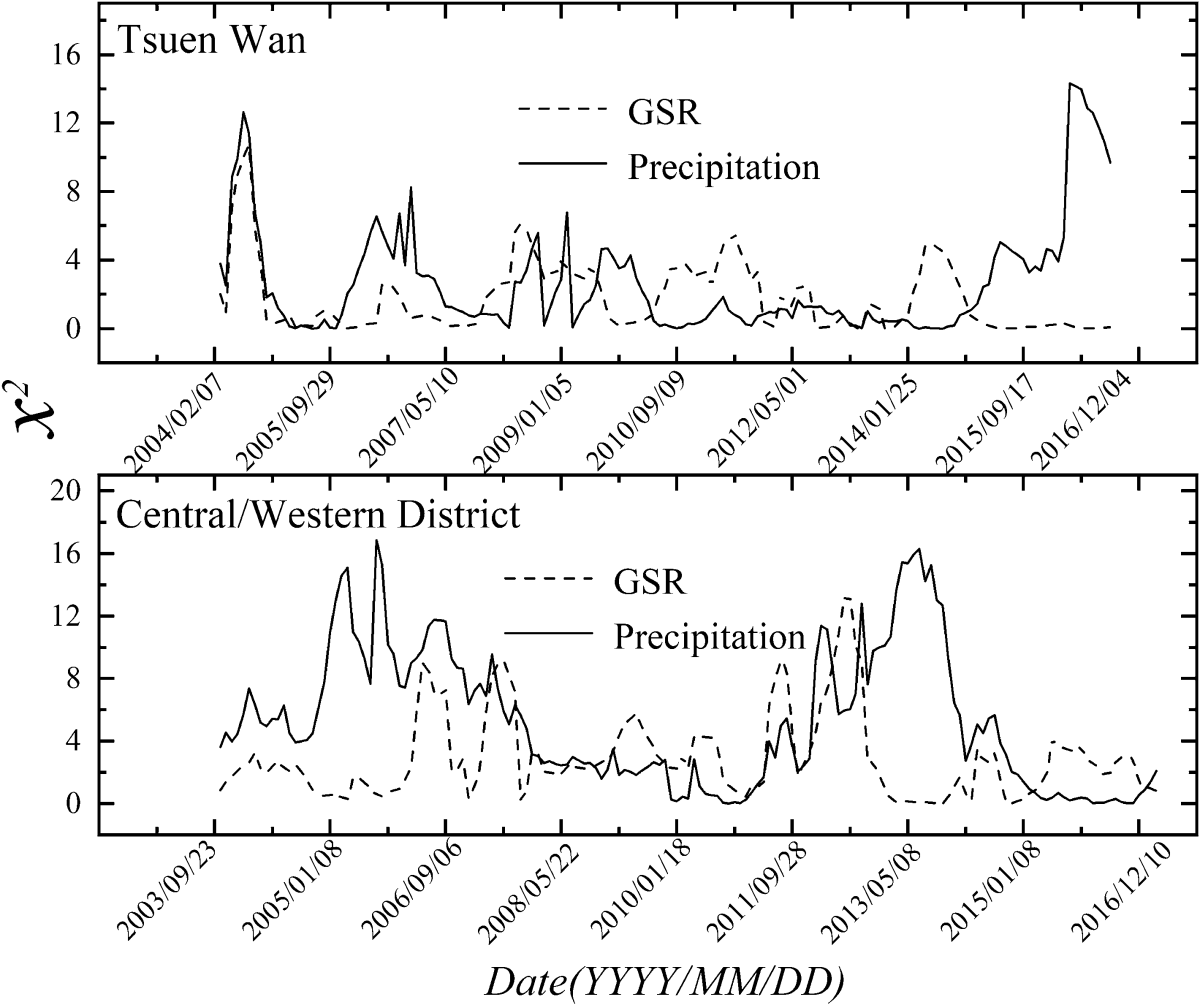
The time dependence of $\chi_{{}}^{2}$ values for precipitation and GSR at the two sites, based on sliding window technique.

In fact, in order to better understand the environmental fate of BaP, our major concern is the temporal evolution of BaP removal rather than that in a certain season. Therefore, it is necessary to describe the temporal distribution of $\chi_{{}}^{2}$ based on sliding window technique. The procedures are as follows: Firstly, set an original window which includes the starting 120 data (about 6 years) of precipitation/GSR and BaP series, and calculate the first $\chi_{{}}^{2}$ value. Then, slide one data towards right keeping the window length unchanged, and calculate the second $\chi_{{}}^{2}$ value. By repeating this procedure, a series of $\chi_{{}}^{2}$ values are obtained over the whole time scale as shown in Fig. 4. It can be observed directly that the inter-annual difference in $\chi_{{}}^{2}$ values was evident for both two sites. Moreover, the peak values of contribution of precipitation and GSR are always not synchronized over the whole time scale. This evidence can be attributed to the fact that in sunny day, there is usually no rain.

**Figure 5:**
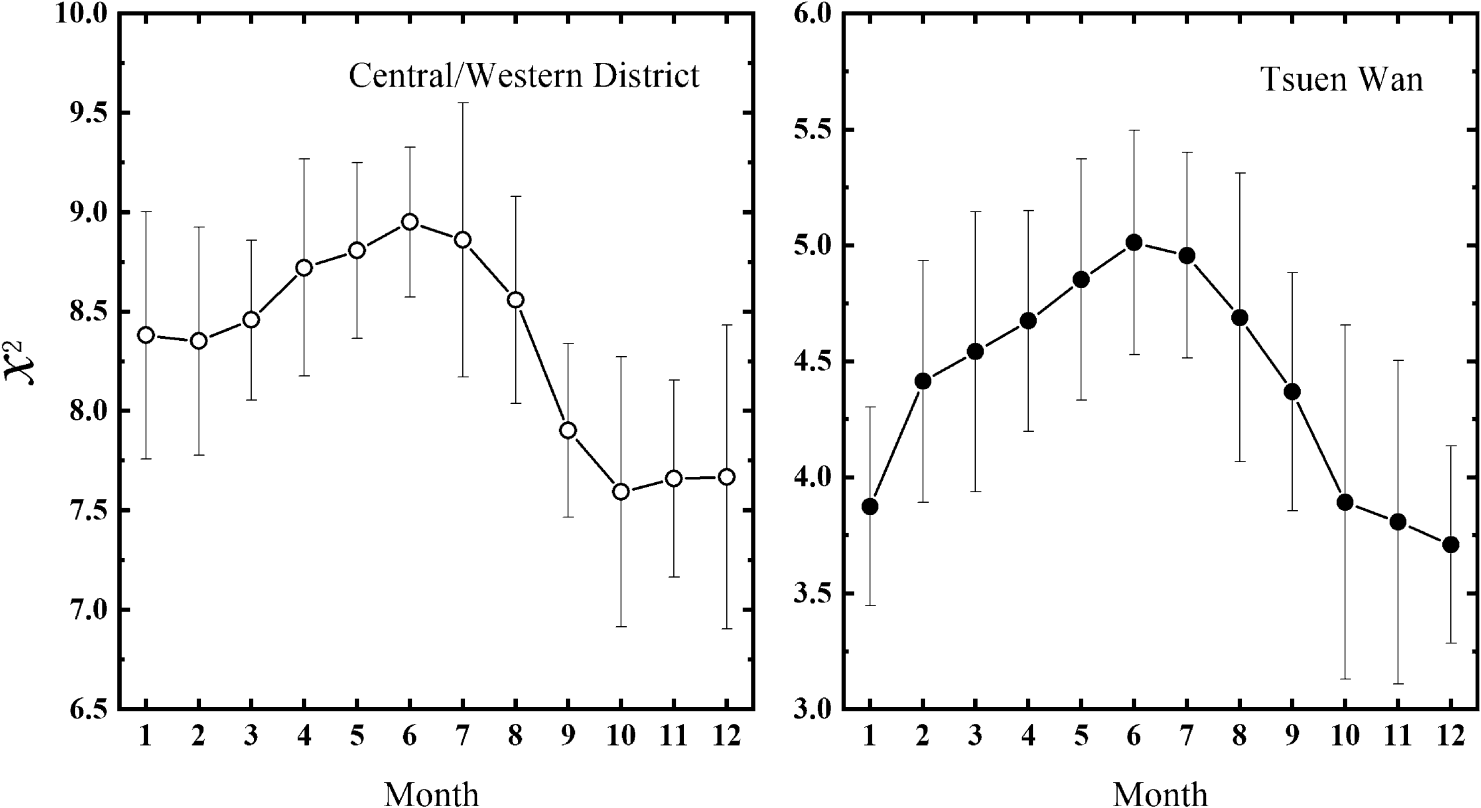
The monthly variations in comprehensive contributions of wet deposition and photodegradation to BaP removal for two sites. Error bars donate the standard deviation.

Finally, to evaluate the comprehensive effects of wet deposition and photodegradation on BaP removal, monthly variations in comprehensive contributions of precipitation and GSR to the removal of atmospheric BaP have been calculated, based on the temporal distributions of the $\chi_{{}}^{2}$ values. Figure 5 shows that monthly variation trend in comprehensive contributions of wet deposition and photodegradation to BaP removal for both Tsuen Wan and Central/Western District sites are consistent. In general, $\chi_{{}}^{2}$ values are higher in May, June and July, and reached its peak in June for both two sites. Then, $\chi_{{}}^{2}$ values decrease gradually and reach the bottom from October to December. These results reveal that the comprehensive contributions of precipitation and GSR are greatest in summer. Moreover, it is worth noting that, besides in summer, the comprehensive contributions are also very great in spring. However, the comprehensive contributions appear relatively weak in autumn and winter. Similar results have been found in other literature. Kong et al. (2010) have found that higher PAHs (BaP) concentrations occurred in January (winter) and October (autumn) in five typical cities of Liaoning Province in China, due to inactive photochemical reactions and insufficient of precipitation^44^.

In this work, MFDCCA analysis provides a new way to quantity the removal efficiency of atmospheric BaP. In future work, it is necessary to further study the effect of meteorological factors on other polycyclic aromatic hydrocarbons (PAHs), especially lower molecular weight PAHs. It is of great significance to evaluate comprehensively the difference in the removal mechanisms of PAHs with different gas-particle partitioning in the atmosphere.

## Conclusions

Based on MFDCCA method, it is found that the cross-correlations between precipitation and BaP present strong long-term persistence within 1-year time scale. But over one year's time scale, there is no correlation between precipitation and BaP. On the other hand, relatively weak persistence has been checked in the cross-correlations between GSR and BaP over the whole time scale. The difference between the results of precipitation-BaP and GSR-BaP system may be related to the meteorology in Hong Kong. The multifractal degree of cross-correlations between precipitation and BaP is much stronger than that between BaP and GSR, which implies that the temporal distribution pattern of cross-correlations between BaP and precipitation are more complex compared with that between GSR and BaP. Furthermore, it is found that the contribution of wet deposition to BaP removal in summer is more than 10 times higher than that in winter. While the contribution of photodegradation to BaP removal in summer is about five times higher than that in winter. Meawhile, the contribution of wet deposition to BaP removal is about two times larger than that of photodegradation in summer. In winter, the difference in the contributions between wet deposition and photodegradation is not significant, and the contribution of photodegradation is a little higher than that of precipitation. Based on the sliding window technique, the temporal evolutions of $\chi_{{}}^{2}$ values for precipitation and GSR show that the inter-annual difference in $\chi_{{}}^{2}$ values was evident for both two sites. On this basis, comprehensive contributions of wet deposition and photodegradation to BaP removal are higher in May, June and July, and reached its peak in June. Then, they decrease gradually and reach the bottom in December.

## Materials and methods

### Materials

**Figure 6:**
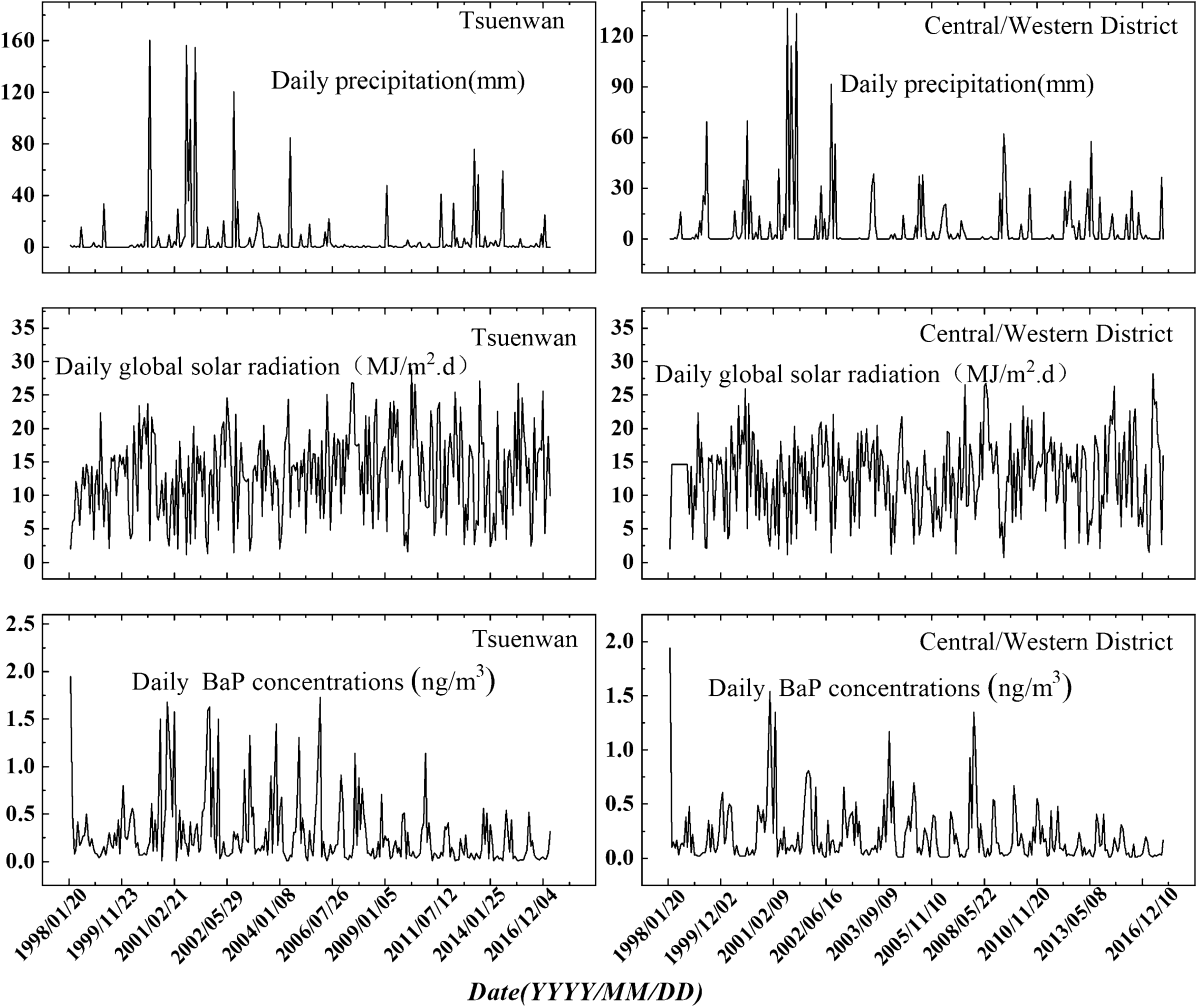
The regular monitoring ambient BaP concentrations, precipitation and GSR of Central/Western District and TsuenWan sites, from January 1998 to December 2016.

Since July 1997, Hong Kong Environmental Protection Department (HKEPD) has set up monitoring facilities for collecting samples of toxic air pollutants at Tsuen Wan and Central/Western District monitoring stations. Tsuen Wan station, located in the southern part of the New Territories in Hong Kong, is a mixed urban residential, commercial and industrial area. While Central/Western District station is located on the northwest coast of Hong Kong Island, which is a mixed urban residential and commercial area. The sampling has been conducted using the TO-3 method recommended by the U.S. Environmental Protection Agency. More detail information about sampling procedures and chemical analysis are available in the previous papers^45^. In short, the ambient BaP samples were collected onto quartz fiber filters, followed by polyurethane foam (PUF) plus XAD-2 resin. The ambient BaP samples were collected once or twice a month from January 1998 to December 2016 by HKEPD. For each sample, the collected period was 24 h. Before GC/MS analysis procedures, the extracts from filter, PUF, and XAD-2 resin were combined. So, ambient BaP concentrations in this paper were the sum of aerosol and gas phase BaP concentrations. Meanwhile, in order to determine the contributions of wet deposition and photodegradation to BaP removal, the synchronized meteorological data including daily precipitation and daily global solar radiation were collected from the Hong Kong Observatory. The observations of ambient BaP, precipitation and GSR for the two monitoring stations are shown in Fig. 6.

### Methods

#### Multifractal detrended cross correlation analysis

The multifractal detrended cross correlation analysis (MFDCCA) has been proposed by Zhou to investigate the multi-fractal features of the coupling correlation between two non-stationary series. The introduction of MFDCCA is as follows:

Firstly, suppose two non-stationary time series *x(t)* and *y(t)*,$t = 1,2, \ldots ,N$,where *N* is the length of the series. Two profiles can be determined as follows:

1 $$ X\left( k \right) = \mathop \sum \limits_{t = 1}^{k} \left[ {x\left( t \right) - \overline{x\left( t \right)} } \right] $$

2 $$ Y\left( k \right) = \mathop \sum \limits_{t = 1}^{k} \left[ {y\left( t \right) - \overline{y\left( t \right)} } \right] $$

where $\overline{{{\text{x}}\left( {\text{t}} \right)}}$ and $\overline{{{\text{y}}\left( {\text{t}} \right)}}$ are the mean values of the two time series.

Secondly, ${\text{X}}\left( {\text{k}} \right)$ and ${\text{Y}}\left( {\text{k}} \right)$ are divided into Ns non-overlapping segments with an time scale s, ${\text{N}}_{{\text{s}}} = {\text{int}}\left( {{\text{N}}/{\text{s}}} \right)$. In fact, the length N is always not a multiple of s, so it is necessary to repeat the same partition method from the other end. In this case, 2Ns segments are obtained together.

Then, for each of the 2Ns segments, the local trend for each segment *v* is calculated using the least-square fitting model. On this basis, the detrended covariance in each segment *v* is determined as follows:

3 $$ f_{xy}^{2} \left( {s,v} \right) = \frac{1}{s}\mathop \sum \limits_{k = 1}^{s} \left[ {X\left( k \right) - \widehat{{X_{v} }}\left( k \right)} \right]\left[ {Y\left( k \right) - \widehat{{Y_{v} }}\left( k \right)} \right] $$

where $v = 1,2, \ldots 2{\text{N}}_{{\text{s}}}$, $\widehat{{{\text{X}}_{{\text{v}}} }}\left( {\text{k}} \right)$ and $\widehat{{{\text{Y}}_{{\text{v}}} }}\left( {\text{k}} \right)$ are the fitting polynomials in segment *v*, respectively.

Next, by squaring and averaging over all segments, the *q*th-moments fluctuation function is calculated:

4 $$ F_{xy}^{q} \left( {q,s} \right) = \left[ {\frac{1}{{2N_{s} }}\mathop \sum \limits_{v = 1}^{{2N_{s} }} \left[ {f_{xy}^{2} \left( {s,v} \right)} \right]^{q/2} } \right]^{1/q} \quad q \ne 0 $$

5 $$ F_{xy}^{0} \left( {q,s} \right) = exp\left\{ {\frac{1}{{4N_{s} }}\mathop \sum \limits_{v = 1}^{{2N_{s} }} ln\left[ {f_{xy}^{2} \left( {s,v} \right)} \right]} \right\} \quad q = 0 $$

Finally, if there is long term power law cross-correlation between the two time series, the *q*th-moments fluctuation function $F_{xy} \left( {q,s} \right)$ will increase with time scale $s$ in the form of power law function.

6 $$ F_{xy} \left( {q,s} \right) \propto s^{{h_{xy} \left( q \right)}} $$

here ${\text{h}}_{{{\text{xy}}}} \left( {\text{q}} \right)$ is generalized cross-correlation exponent, which is defined for describing the power-law cross-correlation relationship between the two time series. For mono-fractal behavior, ${\text{h}}_{{{\text{xy}}}} \left( {\text{q}} \right)$ values are independent of *q*th-moments; while for multi-fractal behavior, ${\text{h}}_{{{\text{xy}}}} \left( {\text{q}} \right)$ values decreases with the increase in *q*th-moments. Moreover, for positive *q*, the corresponding ${\text{h}}_{{{\text{xy}}}} \left( {\text{q}} \right)$ values describe the scaling characteristics of large fluctuations; while for negative *q*,${\text{h}}_{{{\text{xy}}}} \left( {\text{q}} \right)$ describe the scaling characteristics of small fluctuations. It is worth noting that ${\text{h}}_{{{\text{xy}}}} \left( 2 \right)$ is the cross-correlation exponent in DCCA method for *q* = 2. ${\text{h}}_{{{\text{xy}}}} \left( 2 \right) = 0.5$ indicates the absence of cross-correlations between the two time series; ${\text{h}}_{{{\text{xy}}}} \left( 2 \right) > 0.5$ implies the long-term cross-correlations between the two time series; $0 < h_{xy} \left( 2 \right) < 0.5$ indicates the anti-persistent cross-correlations between the two time series.

Additionally, if the cross-correlations between the two time series are multifractal, the multifractality degree of cross-correlation is quantified by an index $\Delta h$,

7 $$ \Delta h = \max \left( {h_{xy} \left( q \right)} \right) - {\text{min}}\left( {h_{xy} \left( q \right)} \right). $$

#### Quantifying the sink of BaP based on MFDCCA

In this work, a new approach is provided to determine the contribution of wet deposition and photodegradation to atmospheric BaP removal based on MFDCCA method. Specific methods are as follows.

Firstly, generalized cross-correlation exponent ${\text{h}}_{{{\text{xy}}}} \left( {\text{q}} \right)$ can be calculated for the original precipitation-BaP and GSR-BaP series based on MFDCCA respectively.

Secondly, the shuffling procedure is applied on the original precipitation/GSR series and kept BaP series unchanged. In this case, the temporal correlations in the data of precipitation/GSR series are destroyed, and the cross-correlation between precipitation/GSR and BaP will disappears. Generalized cross-correlation exponent ${\text{h}}_{{{\text{xy}}}} \left( {\text{q}} \right)$ can be obtained for the shuffled precipitation/ GSR- original BaP series based on MFDCCA respectively. Obviously, the results after shuffling procedure will deviate from the original results. The degree of deviation between the original and shuffled $h_{xy} \left( q \right) \propto q$ curves reflects the contribution of meteorological factors (precipitation and GSR) to the removal of atmospheric BaP.

Thirdly, this deviation of $h_{xy} \left( q \right) \propto q$ curves between the original and shuffled series can be quantified by the chi square $(\chi^{2} )$ test^46^, according to Eqs. (8) and (9). The larger the deviation, the stronger the cross-correlations, and the greater the contribution of precipitation/GSR to BaP removal.

8 $$ \chi^{2} = \sum\limits_{i = 1}^{{N_{q} }} {\frac{{[h(q_{i} ) - h_{shuffled} (q_{i} )]^{2} }}{{\sigma (q_{i} )^{2} + \sigma_{shuffled} (q_{i} )^{2} }}} $$

where *N* is the length of the variable *q.* The mark “shuffled” refers to the obtained results by using the shuffling procedure. $\sigma \left( {q_{i} } \right)$ represents the generalized standard deviation for *q*-th moments , which can be defined as follows:

9 $$ \sigma (q_{i} ) = \left\{ {\frac{1}{N}\sum\limits_{i = 1}^{N} {[(X(i) - \overline{X(i)} )(Y(i) - \overline{Y(i)} )]^{{\frac{{q_{i} }}{2}}} } } \right\}^{{\frac{1}{{q_{i} }}}} $$

here $$\overline{X} = \frac{1}{N}\sum\limits_{i = 1}^{N} {X(i)}$$ and $$\overline{Y} = \frac{1}{N}\sum\limits_{i = 1}^{N} {Y(i)}$$.
